# Supplementary material for: Dynamics of lineage commitment revealed by single-cell transcriptomics of differentiating embryonic stem cells
Source: Nat Commun. 2017 Oct 23;8:1096. doi: 10.1038/s41467-017-01076-4 (PMC5653659; doi:10.1038/s41467-017-01076-4)
Supplement: Supplementary file 1 — Supplementary Information [file 41467_2017_1076_MOESM1_ESM.pdf]

a

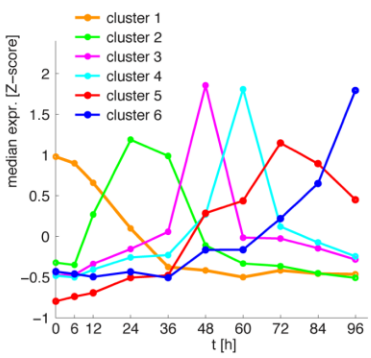

c

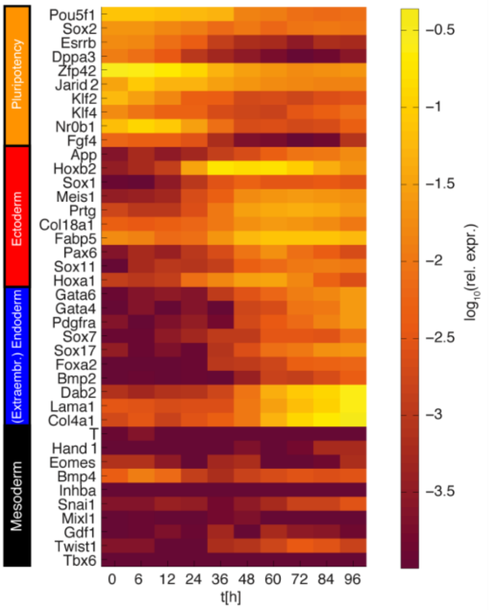

b

cluster 5

GO terms related to development/morphogen./differentiation	q-value
GO:0007507~heart development	1.39E-05
GO:0035107~appendage morphogenesis	1.19E-03
GO:0035108~limb morphogenesis	1.19E-03
GO:0048736~appendage development	1.18E-03
GO:0060173~limb development	1.18E-03
GO:0035113~embryonic appendage morphogenesis	1.47E-03
GO:0030326~embryonic limb morphogenesis	1.47E-03
GO:0035116~embryonic hindlimb morphogenesis	1.76E-03
GO:0035137~hindlimb morphogenesis	6.32E-03
GO:0030900~forebrain development	2.00E-02
GO:0021915~neural tube development	2.06E-02
GO:0009792~embryonic development ending in birth or egg hatching	2.48E-02
GO:0048598~embryonic morphogenesis	2.70E-02
GO:0007389~pattern specification process	2.75E-02
GO:0048538~thymus development	2.88E-02
GO:0043009~chordate embryonic development	3.43E-02
GO:0035239~tube morphogenesis	4.49E-02

cluster 6

GO terms related to development/morphogen./differentiation	q-value
GO:0001568~blood vessel development	6.37E-04
GO:0001701~in utero embryonic development	6.30E-04
GO:0051094~positive regulation of developmental process	1.39E-03
GO:0035295~tube development	3.59E-03
GO:0048514~blood vessel morphogenesis	4.90E-03
GO:0045597~positive regulation of cell differentiation	9.13E-03
GO:0043009~chordate embryonic development	3.22E-02
GO:0009792~embryonic development ending in birth or egg hatching	4.23E-02
GO:0035239~tube morphogenesis	4.52E-02
GO:0001892~embryonic placenta development	4.92E-02

d

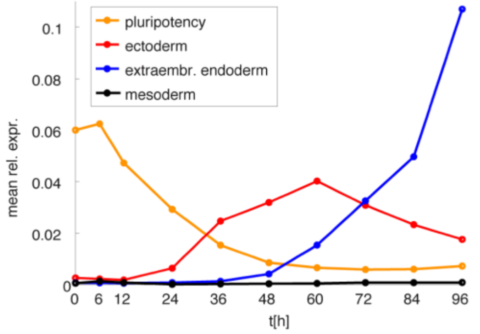

e

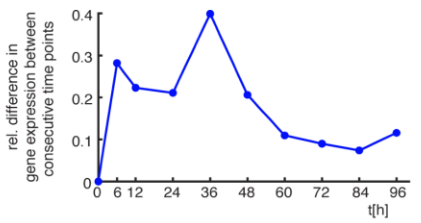

Supplementary figure 1 | Bulk RNA-seq of differentiating mESCs shows downregulation of pluripotency factors and upregulation of germ layer markers

a, Expression profiles measured by bulk RNA-seq were clustered by k-means clustering and 6 robust temporal gene clusters were identified. The median of the expression profiles in each gene cluster is shown. Gene clusters were ordered by the position of the expression maximum. **b**, GO terms which were significantly enriched in clusters 5 and 6 (q-value < 5%). q-values were calculated using the Benjamini-Hochberg procedure from p-values determined by Fisher's exact test. **c**, Gene expression of marker genes for pluripotency and the three germ layers measured by bulk RNA-seq. Expression was normalized to *Gapdh* expression at each time point. **d**, Average expression profiles (relative to *Gapdh*) of marker genes shown in **c. e,** Global gene expression dynamics as measured by bulk RNA-seq. A data point at a certain time point represents the 2-norm of the difference between the gene expression vector at that time point and the previous time point. All differences are normalized to expression at 0 h, which corresponds to cells growing in ground state conditions (2i/L medium)

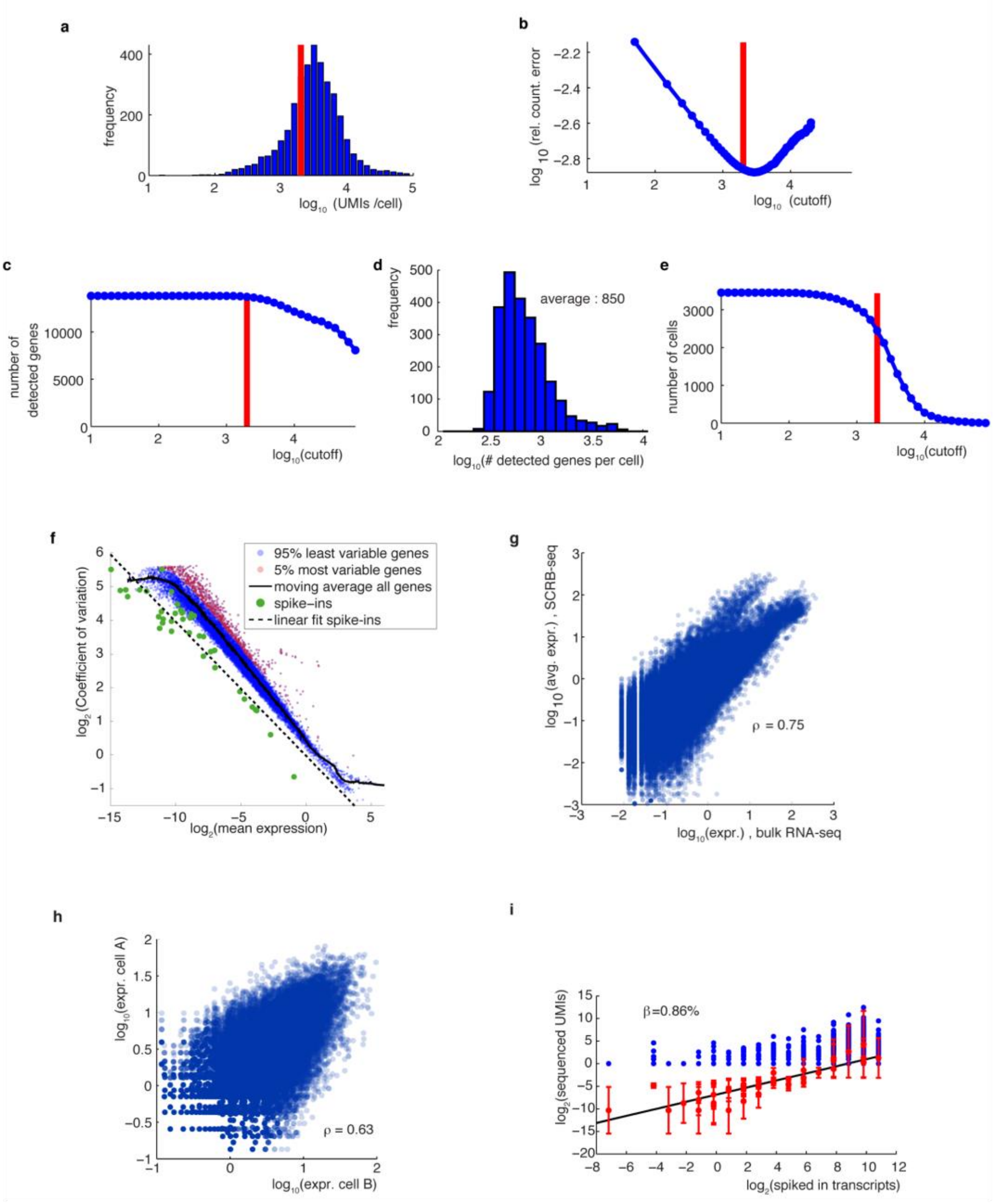

Supplementary figure 2 | Characterization of the SCRB-seq method

a, Frequency of total number of UMIs detected per cell. The red line indicates the cutoff (total UMIs > 2000) above which cells were used for further analysis. **b**, Estimated upper bound for the relative counting error of UMIs with respect to the UMI cutoff. **c**, Number of genes detected across all cells (UMI > 1 in more than 1 cells) with respect to total UMI cutoff. **d**, Distribution of number of detected genes (UMI > 1) per cell for cells with at least 2000 UMIs in total. The average is 850. **e**, Number of cells used for further analysis with respect to total UMI cutoff **f**, Coefficient of variation (CV) of individual genes with respect to mean expression level across all time points. The solid line is a moving average. Indicated in red are the genes, which are considered the 5% most variable taking into consideration the general trend. For comparison, the CVs of spiked in ERCC transcripts are shown in green and a linear fit to these data points is shown as a dashed, black line. **g**, Comparison of bulk RNA-seq measurements and SCRB-seq measurements averaged over cells for individual time points. Pearson correlation ρ = 0.75. **h**, Comparison of expression levels measured by SCRB-seq in 100 randomly selected pairs of single cells in 2i/L conditions. Pearson correlation ρ = 0.63. **i**, Number of spiked in ERCC transcripts with respect to sequenced spike-in UMIs. The blue symbols show the measurements while red symbols indicate the mean; whiskers indicate standard deviations. From a linear fit of the data the recovery efficiency is determined to be 0.9%. The slope of the linear fit is 0.78.

a

PC 1 genes	PC 1 loadings	PC 2 genes	PC 2 loadings
Sparc	0.39	*Prtg*	0.24
Col4a1	0.38	*Ccnd2*	0.23
Lama1	0.35	*Mdk*	0.21
Lamb1	0.33	*Crabp1*	0.19
Col4a2	0.24	*Smoc2*	0.18
Lamc1	0.24	*Mest*	0.17
Serpinh1	0.18	*Peg10*	0.17
Dab2	0.16	*Peg3*	0.16
Lrpap1	0.15	*Frem2*	0.16
Srgn	0.15	*Angptl1*	0.16
Plod2	0.15	*Peg3as*	0.14
Fst	0.13	*Nepn*	0.14
Pth1r	0.13	*Igf2*	0.13
Cryab	0.12	*Fabp5*	0.13
cubn	0.12	*Ednrb*	0.13
Cyp26a1	0.10	*Foxa2*	0.13
F3	0.10	*Tpm1*	0.13
H19	0.09	*Fat3*	0.12
Sox17	0.09	*Nr2f1*	0.12
Gnas	0.09	*Gnas*	0.12
Krt8	0.08	*Cd24a*	0.12
Nrk	0.08	*Malat1*	0.11
Flrt3	0.08	*Id2*	0.11
Adam19	0.07	*Clu*	0.11
Msl3	0.06	*Flrt3*	0.11

b

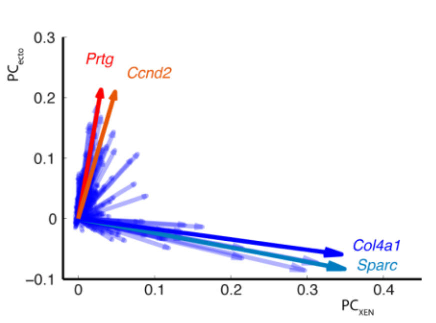

c

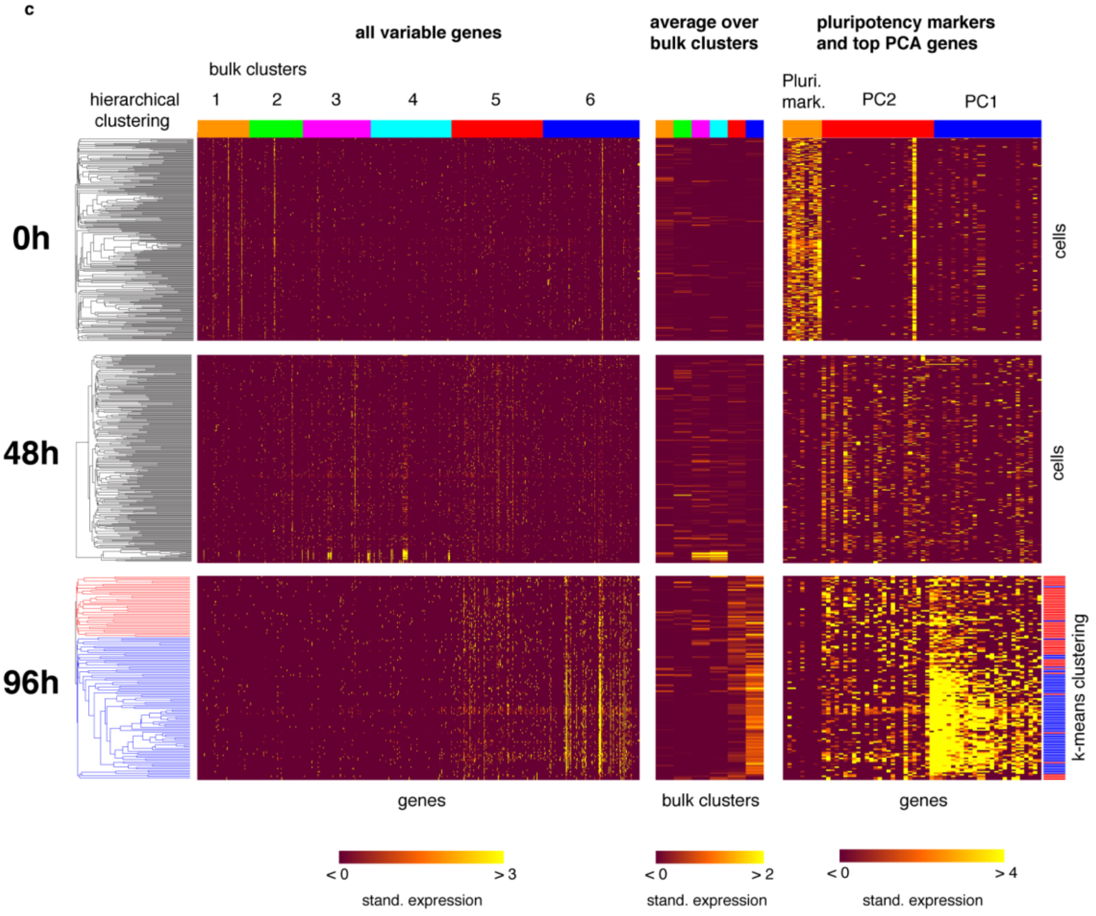

Supplementary figure 3 | Principal component analysis and hierarchical clustering of the SCRB-seq data

a, Loadings of the 25 genes with the highest loadings in the first two principal components (PC 1 and PC 2). Principal components were calculated across all cells and time points. **b**, Graphical representation of the data in **a**. Each arrow represents the loading of a gene. The elements of the vector defining the arrow are the gene's loadings. **c**, Expression levels of the 829 most variable genes at 0 h, 48 h and 96 h of RA exposure. Cells were clustered by complete-linkage clustering using one minus the Pearson correlation as distance metric (dendrograms on the left). The colored lines on the right for the 96h time point indicate k-means clustering, where red corresponds to the ectoderm-like cluster and blue to the XEN-like cluster (see Fig. 1b). The colored bars on top indicate the 6 temporal gene clusters found by bulk RNA-seq (see Supplementary Fig. 1a). In the middle column gene expression was averaged over the genes in the 6 temporal gene clusters. In the rightmost column only the 25 top genes in PC1 and PC2 are shown (see panel **a**) as well as 10 pluripotency markers (*Zfp42, Pou5f1, Klf4, Dppa5a, Esrrb, Sox2, Dppa3, Fgf4, Klf2, Tfcp2l1*). Expression was standardized as described in Methods.

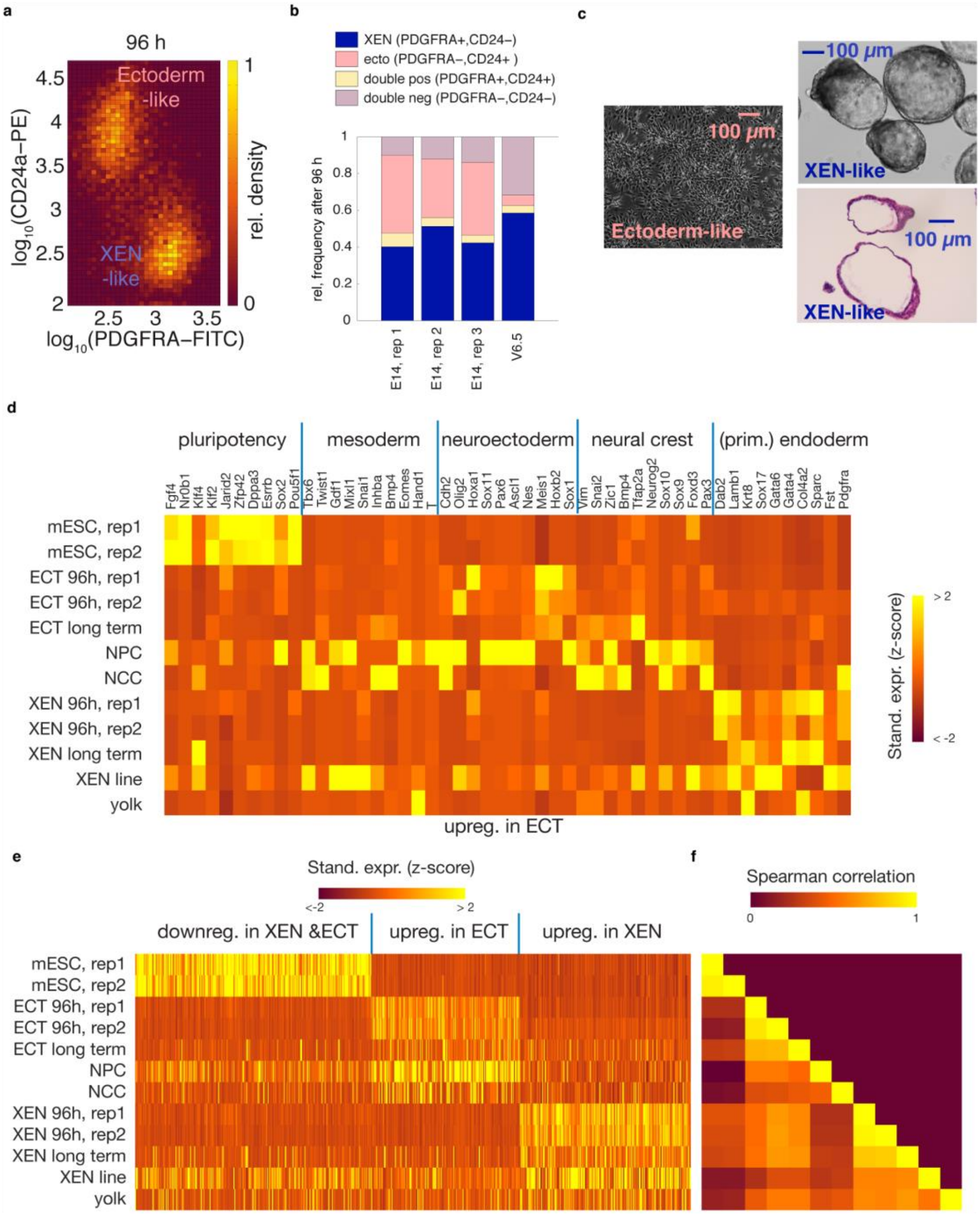

Supplementary figure 4 | Revealing the identity of the differentiated cell clusters by FACS purification and bulk RNA-seq **a**, Flow cytometry measurement of cells stained with CD24 and PDGFRA antibodies after 96 h of RA exposure. The heatmap represents the relative density of cells. **b**, Frequency of XEN-like and ectoderm-like cells after 96 h exposure to RA. Cells were classified based on CD24 and PDGFRA expression measured by antibody staining and flow cytometry. Shown are results for 3 biological replicates with E14 mESCs as well as V6.5 mESCs. **c**, Left panel: ectoderm-like cells (CD24+/PDGFRA-) sorted after 96 h of RA exposure and grown for 5 passages on laminin in N2B27 basal medium complemented with EGF and FGF2. Panels on the right: XEN-like cells (CD24-/PDGFRA+) sorted after 96 h of RA exposure and replated in N2B27 + EGF + FGF2. The top panel shows a phase contrast image of the floating aggregates, which formed quickly after replating. The cyst-like, hollow aggregates shown here were in culture for 3 weeks after sorting. The lower panel is a brightfield image of an H&E stained section of similar floating aggregates, also 3 weeks after sorting. **d,e** Comparison of bulk RNA-seq expression profiles of mESCs, differentiated cells (ECT: ectoderm-like, CD24+/PDGFRA- at 96h; XEN: XEN-like, PDGFRA+/CD24- at

96h), long term cultured purified ECT and XEN cells (see **c**) measured here as well as literature expression data for relevant cell types or tissues (NPC: neural progenitpr cells [1], NCC: neural crest cells [2],yolk: yolk sac [3], XEN line: XEN cell line[4]). In panel **d** a set of marker genes is shown, while panel **e** shows all genes that are differentially expressed between mESCs and XEN and/or ECT. Expression is given as a gene wise z-score to accentuate differences between samples. **f**, Spearman correlation between the expression profiles shown in **e**.

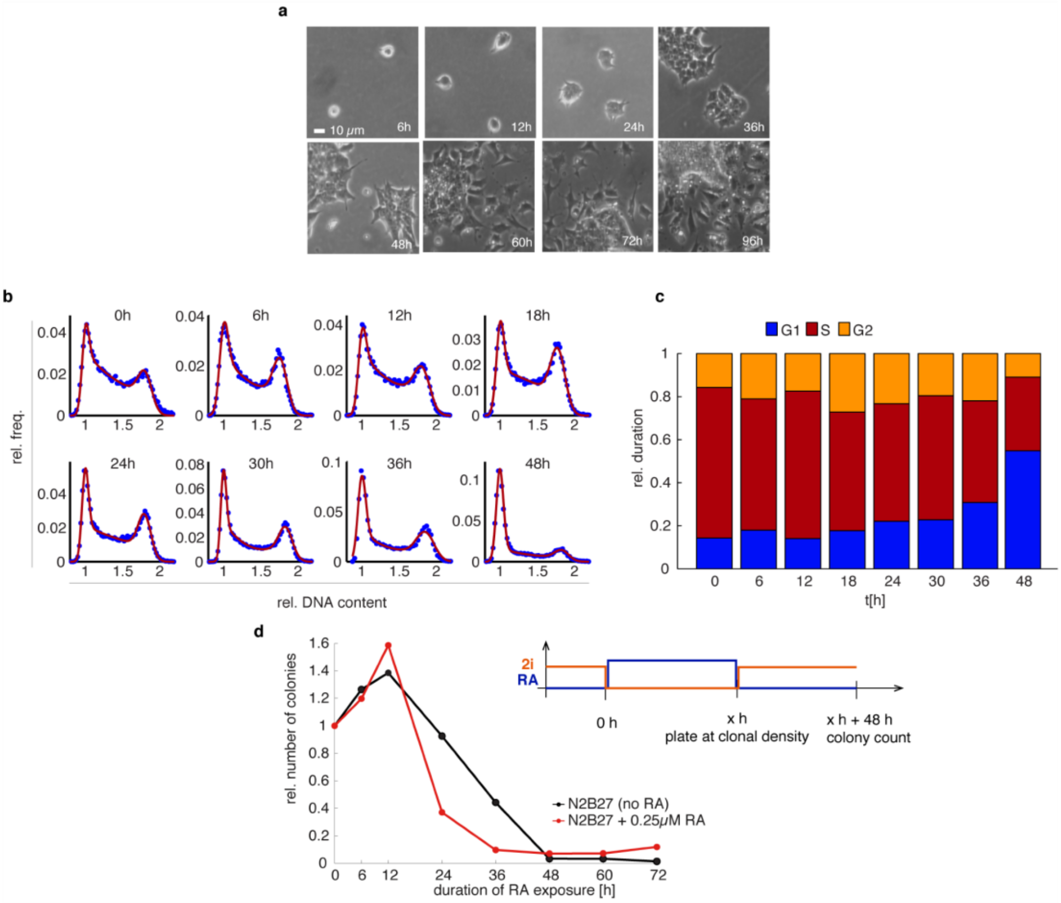

Supplementary figure 5 | Phenotypic characterization of the exit from pluripotency

a, Cell morphology throughout the differentiation time course. Cells were continuously exposed to 0.25 µM RA. Shown are representative phase contrast images. **b**, Histograms of the relative DNA content measured by Hoechst 33342 staining and flow cytometry (blue symbols) after the indicated periods of exposure to RA. The red solid lines are fits of the Dean-Jet-Fox model [5] to the data for individual time points. **c**, Relative lengths of cell cycle phases throughout the time course as determined from fits of the Dean-Jet-Fox model to the measured DNA content. **d**, Clonogenicity after exposure to RA (red data points) or N2B27 basal medium (black data points). After differentiation for the indicated amounts of time cells were replated at a defined, clonal density in 2i/L. After culture for two additional days in 2i/L colonies were counted. The reported values are relative to the number of colonies obtained without differentiation prior to culture in 2i/L. The inset shows a schematic of this experiment.

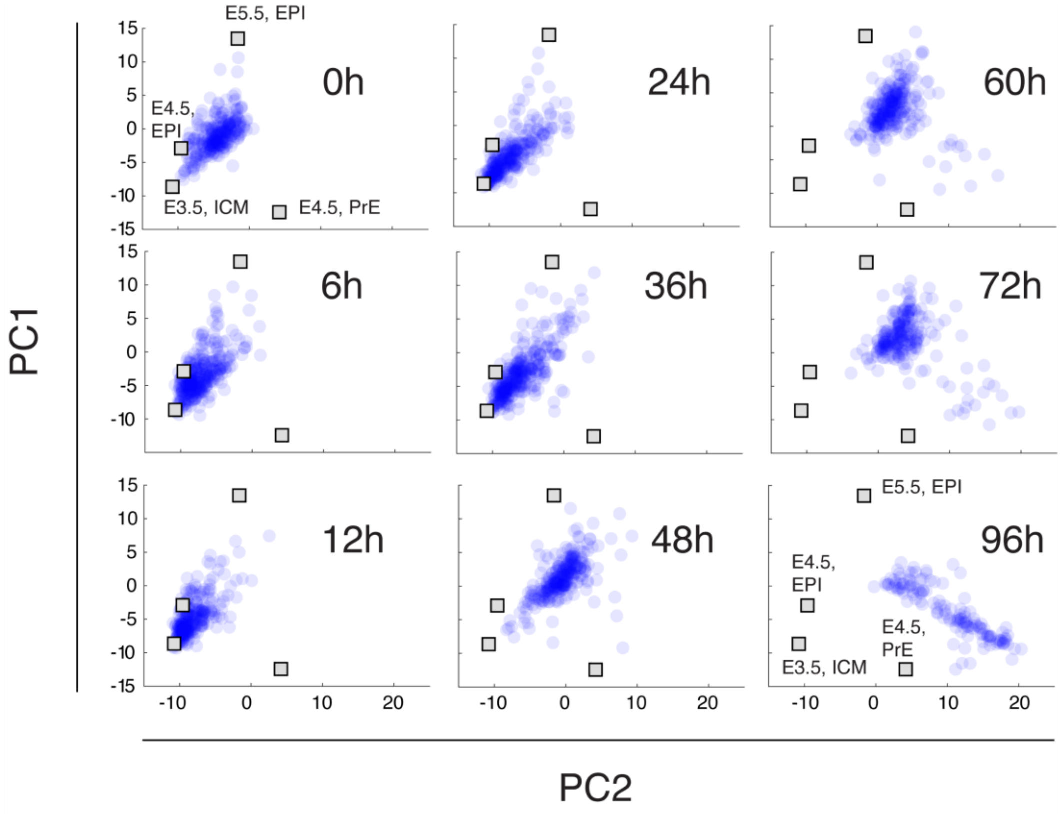

Supplementary figure 6 | Comparison of SCRB-seq data to expression in *in vivo* tissues

Principal component analysis of a panel of pre- / peri-implantation tissues [6]. The SCRB-seq gene expression profiles obtained during RA differentiation were placed in the space of the first two principal components. Each data point represents an individual cell. ICM: inner cell mass, EPI: epiblast, PrE: primitive endoderm

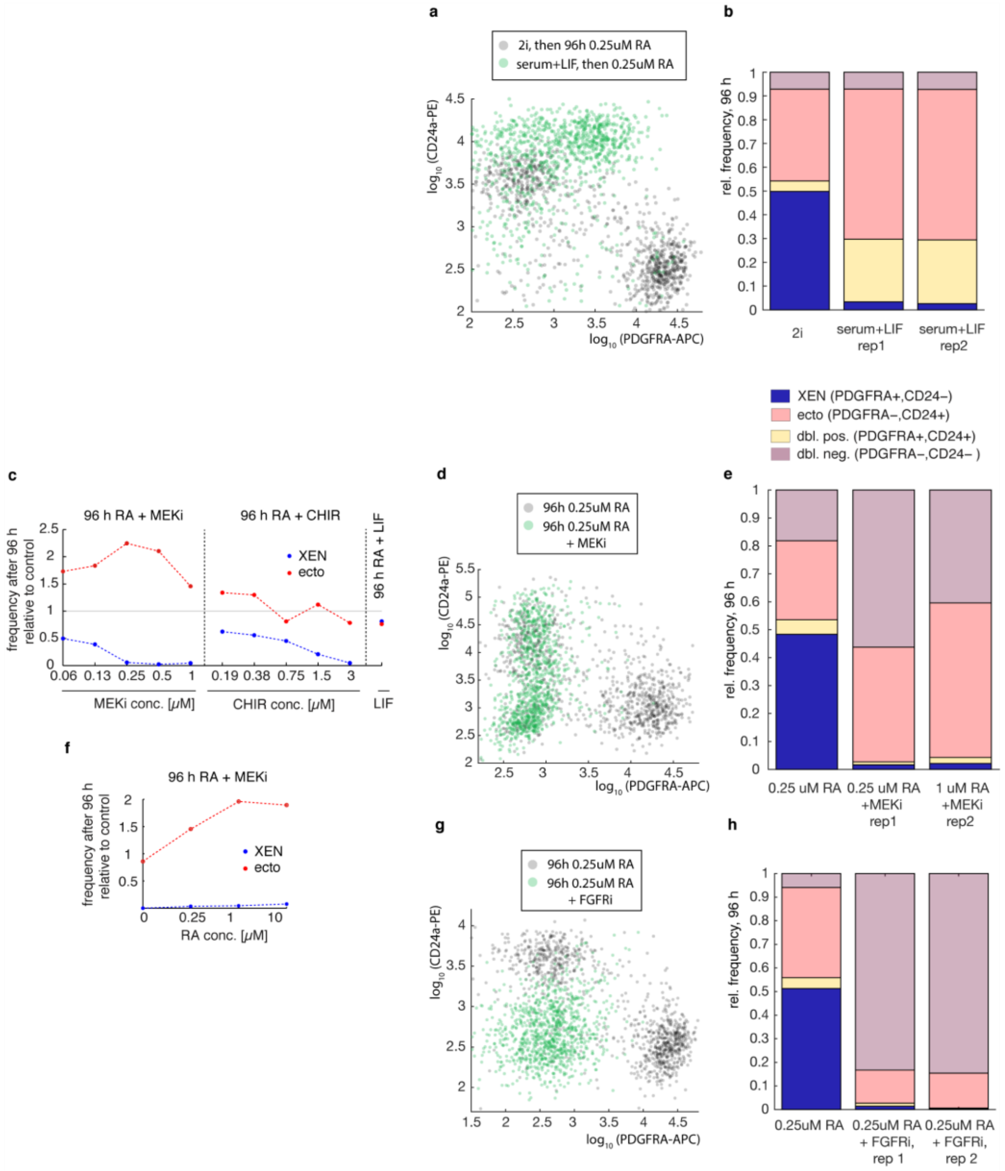

Supplementary figure 7 | Influence of culture conditions, MEK inhibition and FGF receptor inhibition on the lineage decision.

a-h, To determine the frequencies of the various cell types, cells were classified after 96 h of RA exposure based on CD24 and PDGFRA expression measured by antibody staining and flow cytometry. Expression was measured by antibody staining and flow cytometry. **a,** Scatter plot of CD24 and PDGFRA expression after 96 h of RA exposure of cells that were cultured in 2i/L or serum + LIF conditions for 3 passages prior to the start of the differentiation. **b**, Quantification of the experiment shown in **a.** **c**, Frequency of XEN-like and ectoderm-like cells after 96 h differentiation in N2B27 plus RA and MEK inhibitor PD0325901 (MEKi, 0.06 -1 μM), RA and GSK3 inhibitor CHIR99021 (CHIR, 0.19 - 3 μM) or RA and LIF relative to the control (RA only). **d**, Scatter plot of CD24 and PDGFRA expression after 96 h of RA exposure with or without addition of the MEK inhibitor (MEKi) PD0325901. **e**, Quantification of the experiment shown in **d**.

f, Frequency of XEN-like and ectoderm-like cells after 96 h exposure to various concentrations of RA and 0.5 μM MEK inhibitor PD0325901 (MEKi) relative to the control (0.25 μM RA only). **g**, Scatter plot of CD24 and PDGFRA expression after 96h of RA exposure with or without addition of the FGF receptor inhibitor (FGFRi) PD173074. **h**, Quantification of the experiment shown in **g.**

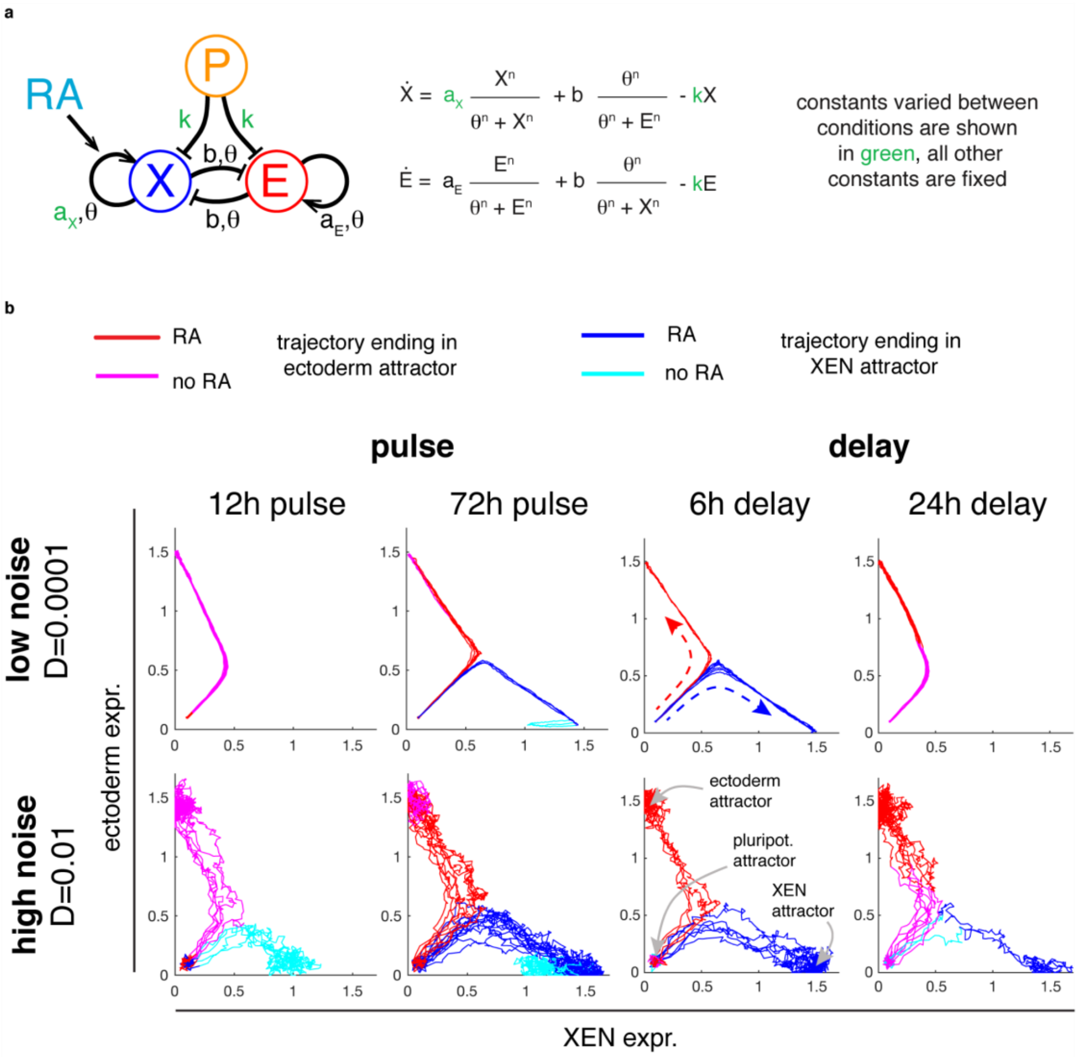

Supplementary figure 8 | Stochastic simulation of the bifurcation process

a, Schematic representation and equations defining a minimal gene regulatory network that can produce a lineage bifurcation [7]. Pointy arrows indicate (auto)activation; blunted arrows indicate repression. E and X represent expression of ectoderm-like and XEN-like transcriptional programs, respectively. P stands for the pluripotency network. Parameters which are changed between conditions are shown in green. All other parameters are fixed. **b**, Exemplary trajectories for the RA pulses or a delay of RA exposure of different lengths. Each panel shows 10 trajectories simulated under the condition indicated at each row and column. All trajectories start at the pluripotency attractor and end either at the XEN or ectoderm attractor (indicated by grey arrows in the “6h delay, high noise” panel). Dashed arrows indicate their overall direction (see the “6h delay, low noise” panel). Trajectories are colored according to their endpoint and the presence of RA. Red-violet trajectories end at the ectoderm attractor, cyan-blue trajectories end at the XEN attractor. During the red and blue parts of trajectories RA is present, during the violet and cyan parts of the trajectories there is no RA.

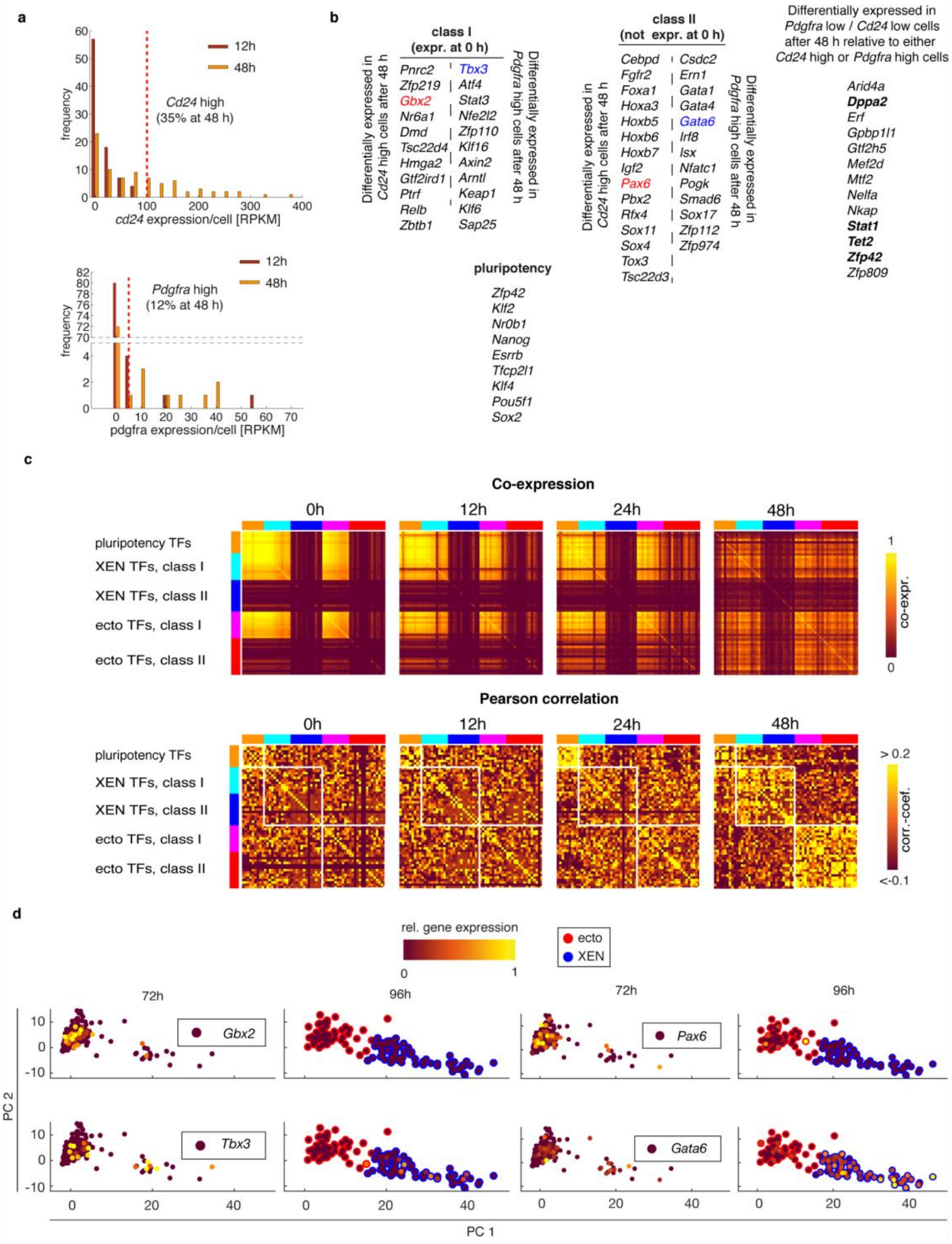

Supplementary figure 9 | Identification of differentially expressed transcription factors with SMART-seq

a, Single-cell expression of *Cd24* and *Pdgfra* after 12 h and 48 h exposure to RA, measured by SMART-seq. The red dashed line indicates the expression level that defines *Cd24* or *Pdgfra* high cells. **b,** List of transcriptional regulators over-expressed after 48 h of RA exposure in *Pdgfra* or *Cd24* high cells. The transcription factors shown in color were picked for further analysis. **c**, Co-expression and Pearson correlation between the transcriptional regulators after 0 h, 12 h, 24 h and 48 h of RA exposure. The gene set comprised the differentially expressed transcriptional regulators identified here (see Fig. 6 a), as well as pluripotency related transcription factors [8] (see **b**). Co-expression and Pearson correlation were calculated using gene expression measured by SMART-seq. Co-expression of two genes was quantified as the fraction of cells with significant expression of both genes. **d**, Single-cell gene expression of *Pax6*, *Gata6*, *Gbx2* and *Tbx3* after 72 h and 96 h of RA exposure, measured with SCRB-seq. The position of each

data point represents the expression profile of a cell in the space of the first two principal components (PC1 and PC2). The color of a data point reflects the expression of the indicated gene, relative to the maximal expression across all cells and the two time points. Cells belonging to the ectoderm-like and an XEN-like cluster after 96 h of RA exposure are indicated by red or blue edges, respectively.

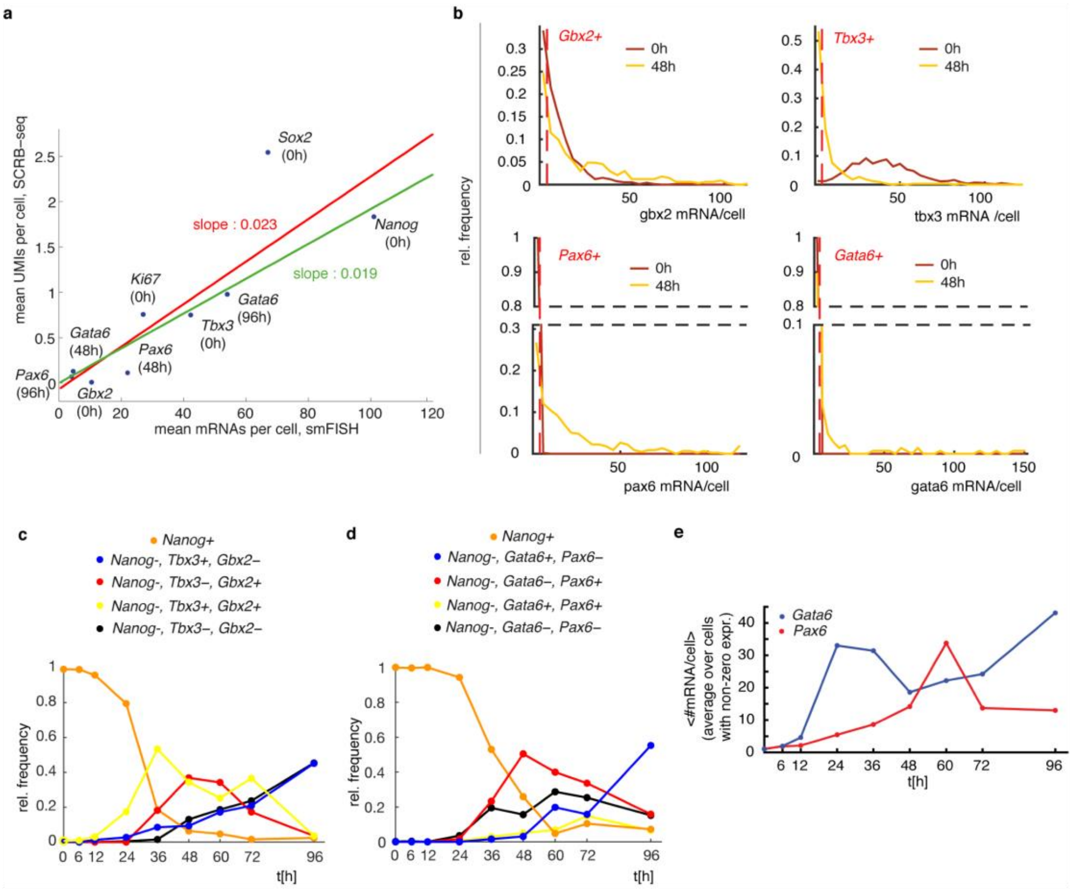

Supplementary figure 10 | Expression dynamics of transcription factors measured by smFISH

a, Sensitivity of smFISH compared to SCRB-seq. Each data point is the single-cell expression level of the indicated gene (at the indicated time point) averaged over all measured cells. The solid lines are linear fits, where the red model had an arbitrary intercept and the green model was forced to go through the origin. smFISH detected approximately 40 times more transcripts than SCRB-seq. **b**, Abundances of *Pax6*, *Gata6*, *Gbx2* and *Tbx3* mRNAs after 0 h and 48 h of RA exposure measured with smFISH. The red dashed line indicates the expression levels that demarcates cells, which are considered positive ("+") for the indicated gene. **c**, Fractions of cells which either expressed *Nanog* or (co)expressed *Gbx2* and *Tbx3* in the absence of *Nanog* throughout the differentiation time course, based on smFISH measurements. Thresholds for significant expression are indicated in **b**. **d**, Fractions of cells which either expressed *Nanog* or (co)expressed *Pax6* and *Gata6* in the absence of *Nanog* throughout the differentiation time course, based on smFISH measurements. Thresholds for significant expression are indicated in **b**. **e**, Number of *Pax6* or *Gata6* transcripts per cell averaged over cells that have at least one *Pax6* or one *Gata6* transcript, respectively. Transcript abundance was measured by smFISH. Cells were exposed to RA for the indicated periods of time.

Supplementary references

1. Wang, J. *et al.* Transcriptome Analysis of Neural Progenitor Cells by a Genetic Dual Reporter Strategy. *STEM CELLS* **29,** 1589–1600 (2011).
2. Hagiwara, K. *et al.* Molecular and cellular features of murine craniofacial and trunk neural crest cells as stem cell-like cells. *PLoS ONE* **9,** e84072 (2014).
3. Stumpo, D. J. *et al.* Deficiency of the placenta- and yolk sac-specific tristetraprolin family member ZFP36L3 identifies likely mRNA targets and an unexpected link to placental iron metabolism. *Development* **143,** 1424–1433 (2016).
4. Julio, M. K.-D. *et al.* Regulation of extra-embryonic endoderm stem cell differentiation by Nodal and Cripto signaling. *Development* **138,** 3885–3895 (2011).
5. Fox, M. H. A model for the computer analysis of synchronous DNA distributions obtained by flow cytometry. *Cytometry* **1,** 71–77 (1980).
6. Boroviak, T. *et al.* Lineage-Specific Profiling Delineates the Emergence and Progression of Naive Pluripotency in Mammalian Embryogenesis. *Developmental cell* **35,** 366–382 (2015).
7. Huang, S., Guo, Y.-P., May, G. & Enver, T. Bifurcation dynamics in lineage-commitment in bipotent progenitor cells. *Developmental biology* **305,** 695–713 (2007).
8. Dunn, S. J., Martello, G., Yordanov, B., Emmott, S. & Smith, A. G. Defining an essential transcription factor program for naive pluripotency. *Science* **344,** 1156–1160 (2014).
